# WIP1 stimulates migration and invasion of salivary adenoid cystic carcinoma by inducing MMP-9 and VEGF-C

**DOI:** 10.18632/oncotarget.3320

**Published:** 2015-03-05

**Authors:** Ya-ling Tang, Xin Liu, Shi-yu Gao, Hao Feng, Ya-ping Jiang, Sha-sha Wang, Jing Yang, Jian Jiang, Xiang-rui Ma, Ya-jie Tang, Yu Chen, Xin-hua Liang

**Affiliations:** ^1^ Department of Oral Pathology, West China Hospital of Stomatology (Sichuan University), Chengdu Sichuan 610041, People's Republic of China; ^2^ State Key Laboratory of Oral Diseases West China Hospital of Stomatology (Sichuan University), Chengdu Sichuan 610041, People's Republic of China; ^3^ Key Laboratory of Fermentation Engineering (Ministry of Education), Hubei University of Technology, Wuhan 430068, People's Republic of China; ^4^ Department of Oral and Maxillofacial Surgery, West China Hospital of Stomatology (Sichuan University), Chengdu Sichuan 610041, People's Republic of China

**Keywords:** wild-type p53 induced phosphatase 1 (WIP1), adenoid cystic carcinoma (ACC), salivary gland, invasion, metastasis

## Abstract

The wild-type p53 induced phosphatase 1 (WIP1) is an oncogene overexpressed in a variety of human cancers. Here, we demonstrated that WIP1 silencing reduced MMP-9 and VEGF-C expression as well as migration and invasion of salivary adenoid cystic carcinoma (ACC) cells. Overexpression of MMP-9 or VEGF-C restored migration and invasion in WIP1 knockdown cells, indicating that MMP-9 and VEGF-C are downstream targets of WIP1 signaling. Levels of cyclin D1 and c-Myc, targets of Wnt/β-catenin pathway, were significantly decreased by WIP1 silencing. In addition, WIP1 expression was positively associated with metastasis and prognosis of ACC patients as well as with MMP-9 or VEGF-C in ACC tissues.

## INTRODUCTION

Adenoid cystic carcinoma (ACC) accounts for roughly 21–24% of salivary gland malignant tumors [1, 2]. Although ACC is characterized by perineural invasion and hematogenous distant metastasis [3, 4], like other tumors, how ACC invade and spread remains a mysterious. Wild-type p53-induced phosphatase 1 (WIP1or PPM1D or PP2Cδ) was a wild type p53-induced Ser/Thr phosphatase and might the only one described with oncogenic function among the type 2C phosphatases till now [5]. WIP1 gene amplification has been explored in 37 of 326 (11.3%) and 27 of 164 (16%) primary breast cancers by Bulavin et al [6] and Li et al [7], respectively. Then, many other groups have verified the WIP1 amplification and overexpression in breast cancers [8, 9], neuroblastoma [10], ovarian clear cell carcinoma [11], medulloblastomas [12], colon cancer [13], nasopharyngeal cancer [14], and non-small cell lung cancer (NSCLC) [15]. Moreover, breast cancers patients with WIP1 amplification had been shown to have a significantly poorer prognosis compared with those without. These data suggest that WIP1 may play a critical role in cancer progression. However, only a fraction of human tumor types have been investigated and it remains to find whether most tumor types, including ACC or only in a subset overexpressed WIP1 protein.

The reports nowadays gave some evidence that WIP1 could be considered as an oncogene and anti-metastatic effector in cancer. For example, Tan et al [16] showed that PPM1D expression was required for the survival of ovarian clear cell carcinoma cell with 17q23.2 amplification. PPM1D overexpression inactivated wild-type p53 and p38 mitogen-activated protein kinase, decreased p16 protein expression, and contributed to cancer malignant progression [9]. WIP1 negatively adjusted the stress responsive p38 mitogen-activated protein kinase (MAPK) pathway by directly inhibiting p38 [17]. PPM1D transcription was controlled by p53 and CREB/ATF family transcription factors [18], PPM1D/WIP1 phosphatase modulated DNA damage signaling induced by the G-quadruplex ligand 12459 [19], and the cyclic thioether peptide (F-pS-I-pY-DDC-amide) can be regarded as PPM1D inhibitor [20]. These data indicated that the involvement of WIP1 in tumor prognosis makes it an attractive drug target for the treatment of cancer, however, the molecular mechanism of the WIP1 in cancer progression should be added.

Herein, we hypothesize that WIP1 promotes the migration and invasion of ACC cells by altering the activity of MMPs and vascular endothelial growth factor (VEGF), which are critical for cancer cells migration and invasion. First, WIP1 expression was examined in a variety of human ACC cell lines by western blot and human tissues by immunohistochemistry. The data showed that WIP1 contributed to ACC migration and invasion. Then, both gain- and loss-of-function studies showed that WIP1 silencing reduced MMP-9 and VEGF-C expression as well as migration and invasion of ACC cells. Overexpression of MMP-9 or VEGF-C restored migration and invasion in WIP1 knockdown cells. We further confirmed that WIP1 exerted its invasion function, at least in part, by promoting Wnt/β-catenin pathway. Our findings show that WIP1 stimulates migration and invasion of salivary adenoid cystic carcinoma by inducing MMP-9 and VEGF-C via activation of the Wnt/β-catenin signaling pathway.

## RESULTS

### WIP1 knockdown inhibits ACC-M cells migration and invasion by modulating MMP-9 and VEGF-C

**Figure 1:**
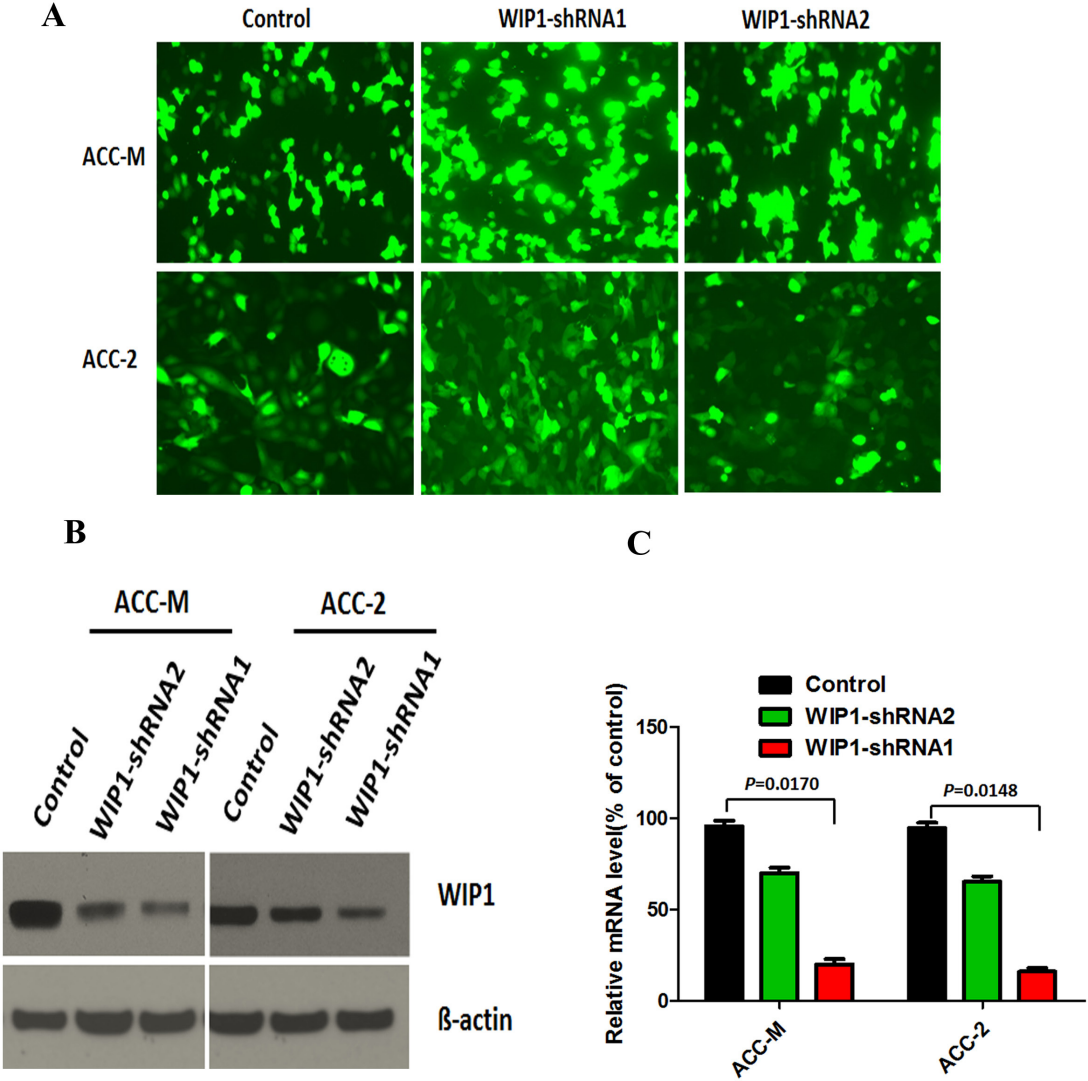
WIP1 silencing in salivary adenoid cystic carcinoma cells **(A)**, ACC-M and ACC-2 salivary adenoid cystic carcinoma cells were transfected with WIP1-shRNA1, WIP2-shRNA2, or control shRNA using Lipofectamine LTX with plus and then photographed under an inverted fluorescence microscope (×100). **(B)**, Western blotting analysis of WIP1 silencing by WIP1-shRNA1 and WIP2-shRNA in both ACC-M and ACC-2 cells. ß-actin loading control is also shown. Representative of three independent experiments was shown. **(C)**, Transcription levels of WIP1 silencing by WIP1-shRNA1 and WIP2-shRNA in both ACC-M and ACC-2 cells, relative to GAPDH, determined by quantitative RT-PCR. Error bars represent the mean ± SD of triplicate experiments.

To explore that the role of WIP1 in the ACC cells *in vitro*, we knock down WIP1 expression by expressing short hairpin RNAs (shRNA). We expressed shRNA from 2 different WIP1 sequences (shRNA1 or shRNA2) in ACC-M and AC-2 cell lines (Figure 1A). Expression of WIP1 protein and mRNA levels were substantially declined by 90% and 85% in WIP1-shRNA1 expressing ACC-M and AC-2 cells, respectively (Figure 1B and 1C). However, the expression of WIP1 protein and mRNA levels were dropped by 20% and 30% in WIP1-shRNA2 ACC-M and AC-2 cells, respectively (Figure 1B and 1C). Thus, 1 of the 2 shRNAs (WIP1-shRNA1) and the 1 of the 2 cell lines (ACC-M) were chosen for further study.

**Figure 2:**
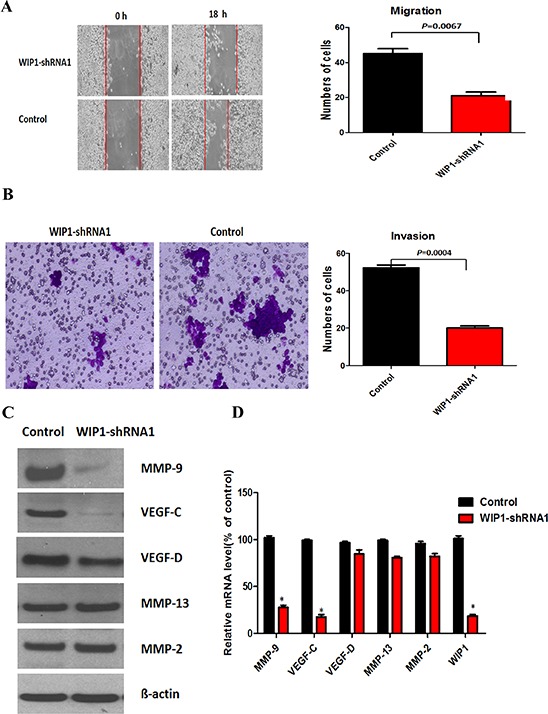
WIP1 silencing inhibits ACC-M cells migration and invasion **(A and B)**, Migration (A) and invasion (B) assays in ACC-M cells. Representative images of migrated and invaded cells were shown under inverted microscopy. The mean was derived from cell counts of 5 fields, and each experiment was repeated 3 times. **(C)**, Western blotting analysis of MMP-9, VEGF-C, MMP-2, MMP-13, and VEGF-D in WIP1-shRNA1 ACC-M cells. ß-actin loading control is also shown. Representative of three independent experiments was shown. **(D)**, Transcription levels of WIP1, MMP-9, VEGF-C, MMP-2, MMP-13, and VEGF-D in WIP1-silenced ACC-M cells, relative to GAPDH, determined by quantitative RT-PCR. Error bars represent the mean ± SD of triplicate experiments.

First we investigated the influence of WIP1 silencing on the cells proliferation and the result showed that WIP1 silencing slightly reduced the proliferative activity of ACC-M cells, compared with the control (Supplementary Figure S1). Then we applied wound-healing assay and transwell invasion assays to investigate the effect of WIP1 silencing on ACC-M cell migration and invasion. Our results showed that WIP1 silencing in ACC-M cells reduced cancer cell migration and invasion at approximately 80% and 85%, respectively, compared with control cells (Figure 2A and 2B). More critically, compared with control cells, the protein and mRNA expressions of MMP-9 were down-regulated by approximately 65% and 70%, respectively, and the protein and mRNA expressions of VEGF-C were declined by approximately 75% and 80%, respectively, in WIP1-silenced cells (Figure 2C and 2D). However, the mRNA and protein expressions of the MMP-2, MMP-13, and VEGF-D did not alter under these conditions.

**Figure 3:**
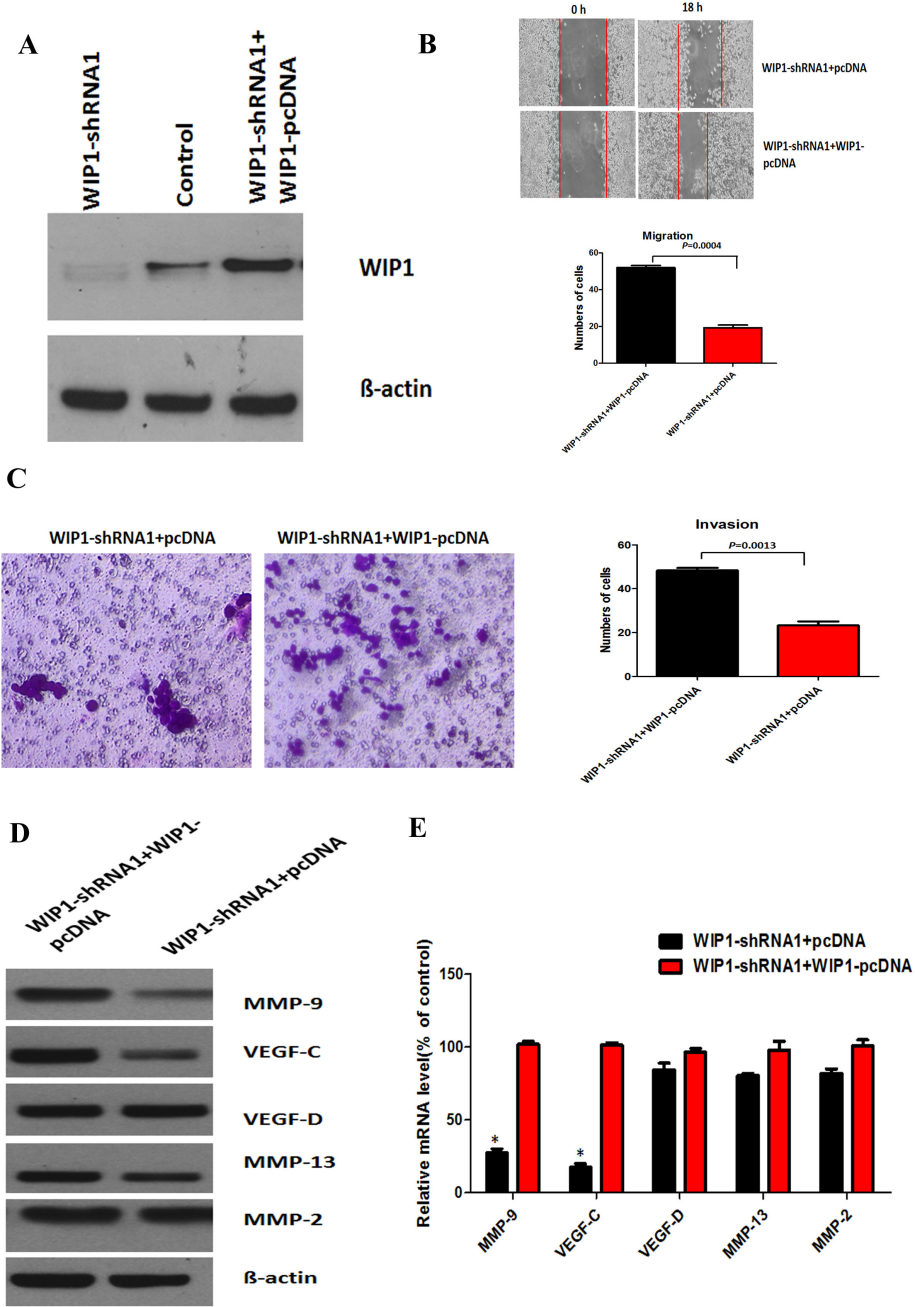
WIP1 modulates cell migration and invasion by regulating MMP-9 and VEGF-C expression **(A)**, Western blotting analysis of WIP1-pcDNA co-cultured with WIP1-shRNA1 ACC-M cells. WIP1 silencing and overexpression were determined by Western blotting. ß-actin loading control is also shown. Representative of three independent experiments was shown. **(B and C)**, The quantitative analysis of migration (B) and invasion (C) in WIP1-pcDNA co-cultured with WIP1 -shRNA1 ACC-M cells. Representative images of migrated and invaded cells were shown under inverted microscopy. The mean was derived from cell counts of 5 fields, and each experiment was repeated 3 times. **(D)**, Western blotting analysis of MMP-9, VEGF-C, MMP-2, MMP-13, and VEGF-D in WIP1-pcDNA co-cultured with WIP1-shRNA1 ACC-M cells. ß-actin loading control is also shown. Representative of three independent experiments was shown. **(E)**, Transcription levels of MMP-9, VEGF-C, MMP-2, MMP-13, and VEGF-D in WIP1-pcDNA co-cultured with WIP1 -shRNA1 ACC-M cells, relative to GAPDH, determined by quantitative RT-PCR. Error bars represent the mean ± SD of triplicate experiments (*p < 0.05).

To further verify whether the effect of WIP1 knockdown on the reduction of migration and invasion of ACC-M cells is unique, we transfected the pcDNA3 plasmid WIP1 vector in WIP1 silencing cells, as confirmed by immunoblotting (Figure 3A) and real-time PCR. We also observed that the up-regulation of WIP1 expression restored ACC-M cell migration and invasion (Figure 3B and 3C) and the protein and mRNA levels of MMP-9 and VEGF-C were added by approximately 65% and 70%, respectively, in WIP1-restored cells, compared with control cells (Figure 3D and 3E). As controls, the protein and mRNA levels of the MMP-2, MMP-13, and VEGF-D did not alter under these conditions.

We have further tested the effects of WIP1 silencing on the migration and invasion of several ACC cell lines (ACC-2, SACC-LM and SACC-83) and found that the migration and invasion of these human salivary gland adenoid cystic carcinoma cells positively correlated with WIP1 expression levels. To further confirm whether WIP1 regulates ACC-M cell migration and invasion by modulating MMP and VEGF expression, we analyzed the protein levels of MMP and VEGF in different cell lines on WIP1 silencing. The Western blotting results showed that the protein levels of MMP-9 and VEGF-C were decreased significantly in WIP1-silenced cells, compared with control cells. As controls, the protein levels of the MMP-2, MMP-13, and VEGF-D did not alter on WIP1 silencing. These results showed that the expression levels of WIP1 positively correlated with the migration and invasion of human salivary gland adenoid cystic carcinoma cells.

### MMP-9 and VEGF-C are downstream targets of WIP1

**Figure 4:**
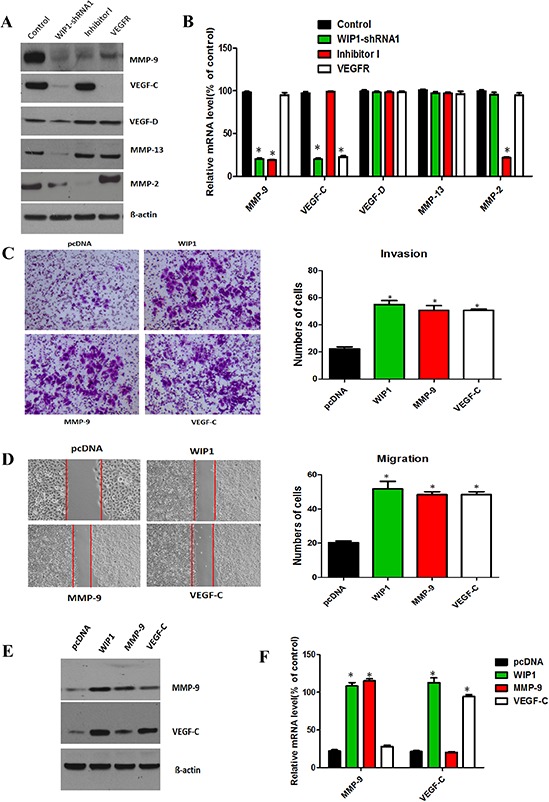
MMP-9 and VEGF-C are downstream targets of WIP1 **(A)**, Western blotting analysis of MMP-9, MMP-2, MMP-13, VEGF-C and VEGF-D on WIP1 silencing and inhibitor I and VEGFR-3 in ACC-M cells. ß-actin loading control is also shown. Representative of three independent experiments was shown. **(B)**, The mRNA levels of MMP-9, MMP-2, MMP-13, VEGF-C and VEGF-D were quantified by real-time PCR on WIP1 silencing and MMP inhibitor and VEGFR compared with control cells. GAPDH mRNA levels were used as internal controls. Error bars represent the mean ± SD of triplicate experiments (*p < 0.05). **(C and D)**, Decreased migration and invasion in WIP1 knockdown ACC-M cells were rescued by MMP-9 and VEGF-C overexpression. The quantitative analysis of invasion (C) and migration (D) in WIP1 or MMP-9 or VEGF-C expression vector cells co-transfected with WIP1-shRNA1. The quantitative analysis of migration was measured at 18 h after cell culture. Representative images of migrated and invaded cells were shown under inverted microscopy. The mean was derived from cell counts of 5 fields, and each experiment was repeated 3 times (*p < 0.05). **(E)**, Western blotting analysis of the protein levels of MMP-9 and VEGF-C on ectopic expression of WIP1 or MMP-9 or VEGF-C in WIP1 or MMP-9 or VEGF-C expression vector cells co-transfected with WIP1-shRNA1. **(F)**, RT-PCR analysis of the mRNA levels of MMP-9 and VEGF-C on ectopic expression of WIP1 or MMP-9 or VEGF-C. GAPDH mRNA levels were used as internal controls. Error bars represent the mean ± SD of triplicate experiments (*p < 0.05).

To further investigate whether MMP-9 and VEGF-C activity is necessary for the migration and invasion of ACC-M cell, we examined the influence of inhibitor I, a specific inhibitor of MMP-2 and MMP-9, and the receptor of VEGF-C (VEGFR-3, 200 ug/mL, 100 uL) on cells. Both inhibitors significantly inhibited ACC-M cells migration and invasion (Supplementary Figure S2A, S2B), showing that MMP-9 and VEGF-C expression is demanded for ACC-M cells migration and invasion (Supplementary Figure S2A, S2B), showing that MMP-9. Our previous data have showed that the mRNA and protein levels of MMP-9 and VEGF-C were decreased significantly in WIP1-silenced cells, respectively, compared with the control (Figure 2C and 2D). Herein, we investigated the protein levels of MMP-9 and VEGF-C in the inhibitors cells. The protein expression of MMP-9 and VEGF-C was reduced by approximately 80% and 70%, respectively, in inhibitor cells, compared with control cells. The inhibition effect of protein expression of MMP-9 and VEGF-C on WIP1 silencing was similar with the inhibitors, indicating that WIP1 participates in the transcriptional regulation of MMP-9 and VEGF-C in ACC-M cells (Figure 4A and 4B).

In addition, we stably overexpressed WIP1 to restore WIP1 expression and found that the overexpression of WIP1 significantly regained mRNA levels of MMP-9 and VEGF-C in WIP1silencing cells (Figure 3E). Then, we showed that MMP-9 or VEGF-C overexpression successfully rescued the capacities of migration and invasion in WIP1-silenced cells (Figure 4C and 4D). We also detected the protein and mRNA levels of MMP-9 and VEGF-C during this process. The restoration of expression of MMP-9 or VEGF-C increased the migration and invasiveness of WIP1-silenced cells. Furthermore, the overexpression of WIP1 restored protein and mRNA levels of MMP-9 or VEGF-C in WIP1-silenced cells (Figure 4E and 4F) and significantly increased the migration and invasiveness of these cells, indicating MMP-9 and VEGF-C are downstream targets of WIP1.

**Figure 5:**
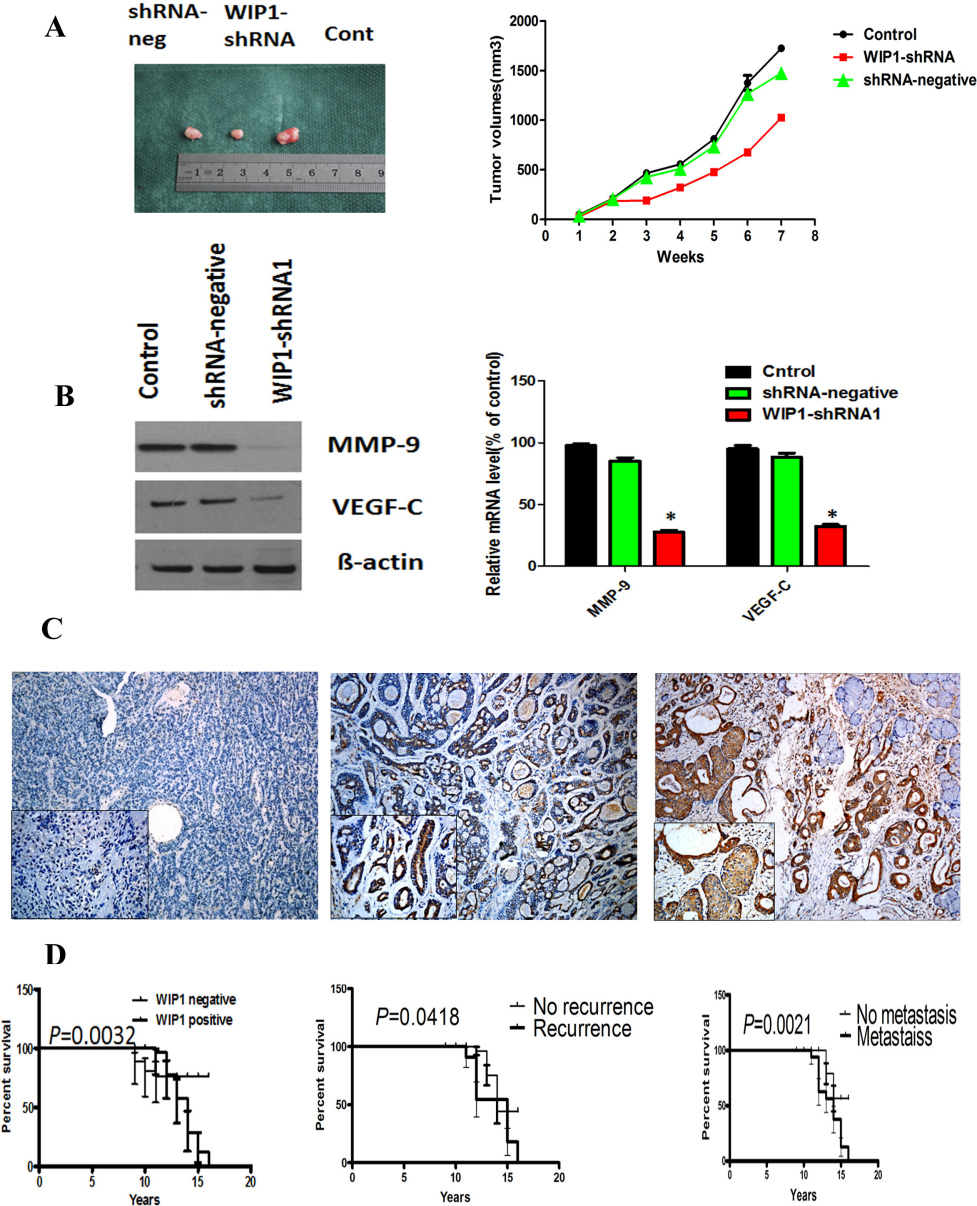
MMP-9 and VEGF-C were downstream targets of WIP1 *in vivo* and WIP1 expression was associated with the poorer prognosis of ACC patients **(A)**, Stable WIP1-shRNA1 ACC-M cells were subcutaneously injected into nude mice. Individual tumor volume was measured at the 7th week after injection and growth curve of xenograft tumors was shown. **(B)**, Western blotting and RT-PCR analysis of the protein and mRNA levels of MMP-9 and VEGF-C in shRNA-neg, WIP1-shRNA1 and control group. Error bars represent the mean ± SD of triplicate experiments (*p < 0.05). **(C)**, WIP1 expression was associated with invasive subtypes of human ACC. Representative images of the immunohistochemical staining of WIP1 in ACC samples. C left, WIP1 in normal human salivary tissue. C middle, WIP1 in weak tumor staining. C right, WIP1 in strong tumor staining. Original magnification, × 100; inset, × 200; bar, 100 mm. **(D)**, Kaplan-Meier survival analysis in patients with ACC. Overexpression of WIP1 in ACC was associated with a shorter overall survival in the respective group.

Finally, we established xenograft using cells treated respectively with shRNA-neg, shRNA1 and control, and documented the tumor volume weekly. As shown in Figure 5A right, there was no difference of tumor volume between the shRNA1 and the shRNA-neg groups in the first two weeks, but the growth of the tumor in the shRNA1 group significantly slowed down since the 4th week, compared with the shRNA-neg group (*p* < 0.05).

We harvested the tumors at the 7th week (Figure 5A left), and then evaluated the proliferation of the tumors. The ki-67 immunohistochemical stainings in the shRNA1-treated tumors were remarkably lower than that in shRNA-neg-treated tumors, indicating that interference for WIP1 may restrain the proliferation of tumor cells. The protein and mRNA expression levels of MMP-9 and VEGF-C were determined by Western blot and RT-PCR in each group. The results showed that the protein and mRNA levels of MMP-9 and VEGF-C were significantly lower in shRNA1-WIP1 cells than shRNA-neg and control group (Figure 5B). The data indicated that MMP-9 and VEGF-C were downstream targets of WIP1.

### Correlation between WIP1, MMP-9 and VEGF-C expression and clinicopathologic factors in ACC cases

To investigate the clinical role of WIP1 expression in ACC cases, we carried out immunohistochemistry staining of WIP1 in 121 human ACC samples representing different histological patterns, 20 specimens of pleomorphic adenomas and 10 normal human salivary gland tissues. Representative immunohistochemical images were shown in Figure 5C. We found that WIP1 was expressed in 67.77% (82/121) specimens of salivary ACC, majorly located in the cytoplasm and occasionally nuclei. Three specimen of 20 pleomorphic adenomas showed WIP1 positive expression and the normal salivary gland tissue had no WIP positive expression. Salivary ACC had more WIP1 expression compared with the other two groups (*p* < 0.05).

The relationship between the expression of WIP 1 and clinicopathologic features of ACC was shown in Table 1. There was significant difference of WIP positive expression between tubular or cribiform pattern and solid pattern in ACC (*p* = 0. 0196). The rate of WIP1 positive expression in patients with invasion, recurrence and metastasis was much higher, compared with negative patients (*p* = 0.0910, *p* = 0.0430, *p* = 0.0262, respectively). However, WIP1 positive expression status did not associate with age, sex, resection margins and complaints of all patients (*p* > 0.05).

**Table 1:** Clinicopathologic features of the ACC patients and their primary tumors and their association with WIP1 expression (*n* = 121)

Clinicopathological features	No. of cases	WIP1 expression		*P*
		Negative	Positive	
Age (years) at diagnosis	121			1.000
<50	45	14	31	
>=50	76	25	51	
Sex	121			0.8130
Female	67	17	50	
Male	54	22	32	
Complaints, months	121			0.1190
<12	64	25	39	
≥12	57	14	43	
Site	121			0.0017
Minor salivary gland	63	12	51	
Major salivary gland	58	27	31	
Histological subtype	121			0.0196
Tubular/Cribiform	85	33	52	
Solid	36	6	30	
Resection margins	121			0.8305
Free	88	29	59	
Affected	33	10	23	
Perineural invasion	100			0.0910
Yes	47	8	39	
No	53	22	31	
Local regional recurrence	121			0.0430
Yes	30	5	25	
No	91	34	57	
Distant metastasis	103			0.0262
Yes	39	6	33	
No	64	23	41	

Survival curves showed that the positive WIP1 or metastasis patients showed a lower survival rate than with negative WIP1 (*p* = 0.0032, Figure 5D). The univariate and multivariate analyses in all the patients demonstrated that WIP1 expression, recurrence and metastasis were independent and significant prognostic clinic-pathologic features (*p* < 0.05). These observations implicate the potential usefulness of WIP1 as a novel prognostic molecular marker for ACC. Simultaneously, the ACC specimens were immunostained for MMP-9 and VEGF-C. The positive expression of WIP1 significantly associated with MMP-9 or VEGF-C positive expression in ACC (*p* < 0.05).

### Wnt/ß-catenin plays an important role in WIP1 regulation of the activity of MMP-9 and VEGF-C

**Figure 6:**
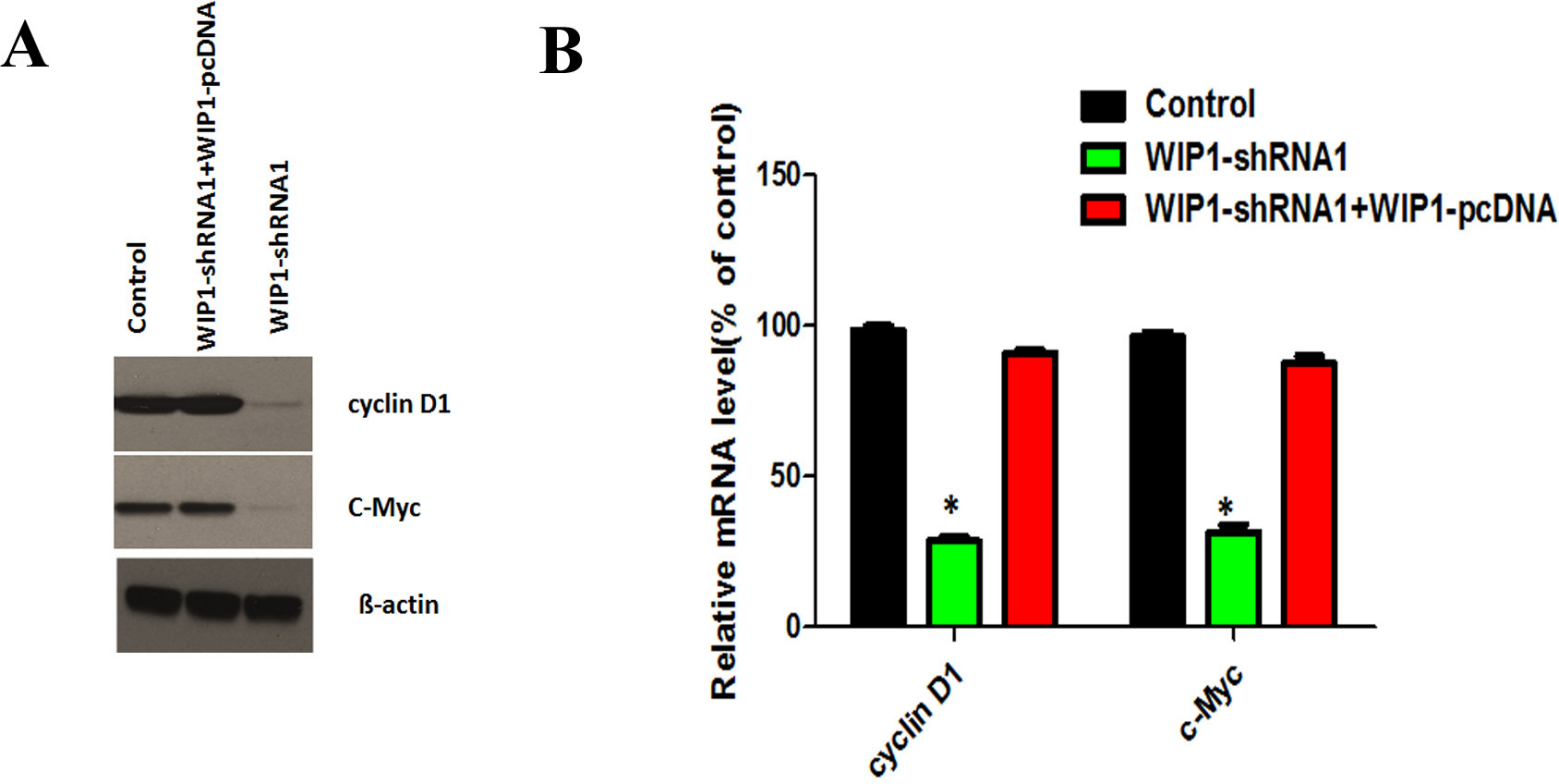
WIP1 silencing decreased the expression of cyclin D1 and c-Myc **(A)**. Effects of WIP1 silencing on the protein levels of cyclin D1 and c-Myc, the known target genes of Wnt/β-catenin pathway, were analyzed by Western blotting after control and WIP1-shRNA1with or without WIP1 overexpression vector, were co-cultured with ACC-M cells. ß-actin loading control is also shown. Representative of three independent experiments was shown. **(B)**, Effects of WIP1 silencing on the mRNA levels of cyclin D1 and c-Myc were analyzed by RT-PCR after control and WIP1-shRNA1with or without WIP1 overexpression vector, were co-cultured with ACC-M cells. GAPDH mRNA levels were used as internal controls. Error bars represent the mean ± SD of triplicate experiments (*p < 0.05).

WIP1 has been reported to regulate neurogenesis during aging via DKK3-dependent regulation of Wnt [21] and play an important role in Wnt signaling pathways [15]. To reveal the signaling pathways that WIP1 regulates MMP-9 and VEGF-C expression, we investigated the effects of WIP1 knockdown on the expression of Wnt/ß-catenin signaling pathway. We found that the protein and mRNA levels of cyclin D1 and c-Myc, the recognized targets of Wnt/β-catenin pathway [22], were significantly decreased in WIP1-silenced ACC-M cells, which was restored by overexpressing WIP1 (Figure 6A and 6B). These results suggested that WIP1 regulates MMP-9 and VEGF-C expression and promotes ACC-M cells migration and invasion at least partly by facilitating Wnt/ß-catenin signaling.

## DISCUSSION

This study identifies WIP1 as a marker of metastasis and poorer prognosis of ACC patients. We demonstrated that WIP1 silencing reduced MMP-9 and VEGF-C expression and the migration and invasion of ACC cells, and we also proved that overexpression of MMP-9 or VEGF-C restored the migration and invasion in WIP1 knockdown cells, indicating that MMP-9 and VEGF-C are downstream targets of WIP1 signaling. In addition, we found that levels of cyclin D1 and c-Myc, targets of Wnt/β-catenin pathway, were significantly decreased by WIP1 silencing. These indicated that WIP1 can regulate MMP-9 and VEGF-C expression through activation of the Wnt/β-catenin signaling pathway to promote ACC migration and invasion.

The first evidence of a role for WIP1 contributing to cancer progression was that we found WIP1 silencing decreased the migration and invasion of ACC-M cells, which was in line with many previous reports. Fu et al [15] demonstrated that WIP1 siRNA had a lower cell proliferation and a higher cell apoptosis in NSCLC. Buss et al [23] found that WIP1 knockdown inhibited medulloblastoma growth and invasion. Zhang et al [14] showed that WIP1 regulates the proliferation and invasiveness of nasopharyngeal carcinoma (NPC) cells *in vitro*, and this may be correlated with its modulation of MMP-9 expression, cell cycle progression and apoptosis. Importantly, we restored the migration and invasion of WIP1 silencing ACC-M cells by stably overexpressing WIP1, indicating that WIP1 promoted cancer cell migration and invasion.

To further confirm that WIP1 accelerated cancer progression *in vitro* and *in vivo*, we delineated the correlation between WIP1 and MMP-9 and VEGF-C to determine how WIP1 promoted ACC-M cells migration and invasion. MMP-9, an important member of the matrix metalloproteinase family, plays a critical role in extracellular matrix degradation, invasion and metastasis in basal-like triple negative breast cancer [24], gastric cancer [25] and glioma [26]. WIP1 regulates the proliferation and invasiveness of NPC cells *in vitro*, and this may be correlated with its modulation of MMP-9 expression [14]. In the previous study, we have showed that the level of MMPs and VEGF have been shown to positively correlate with patients progression in oral squamous cell carcinoma (OSCC) [27]. The present study showed that it was the mRNA and protein levels of MMP-9 and VEGF-C, but not MMP-2, MMP-13, and VEGF-D that WIP1 silencing significantly inhibited, and that the up-regulation of MMP-9 or VEGF-C successfully rescued the migration and invasion of WIP1 silencing ACC-M cells. These indicated that WIP1 specifically regulated MMP-9 and VEGF-C.

Then, the signal transduction signaling that adjust the activity of MMP-9 and VEGF-C transcription factors are examined. Not only delta-beta-catenin expression significantly added the levels of MMP-2, MMP-3, MMP-7, MMP-9, MT3-MMP, and ADAMTS5 [28–30], but beta-catenin was also regarded as a critical effector of E-cadherin disruption up-regulating VEGF-A and VEGF-C [31]. This study showed that the knockdown of WIP1 expression by shRNA could significantly reduce the levels of cyclin D1 and c-Myc, the important target genes of the Wnt/β-catenin pathway. Thus, Wnt/β-catenin pathway has involved in WIP1 regulating the transcriptional levels of MMP-9 and VEGF-C in ACC invasion and metastasis.

Finally, we found that WIP1 expression was showed in 82 of all tumors analyzed, where 9 cases were located in cell nuclear, and 73 cases located in cytoplasmic. The observation is consistent with other publications [10–12]. However, immunohistochemistry demonstrated a mainly nuclear expression of PPM1D in colorectal cancer [32]. Much work should be done to illuminate the reason and significance of cytoplasm staining of WIP1. The relationship between WIP1 expression and clinicopathologic parameters in ACC patients indicated that WIP1 correlated with malignant prognosis of salivary adenoid cystic carcinoma: 1) Fu et al [15] and Peng et al [32] found that the normal salivary gland tissue had no WIP1 positive expression. Herein, we obtained the similar data in normal salivary gland tissue and found that ACC specimens possessed more WIP1 protein compared with benign tumors and normal salivary gland tissue. 2) Reportedly, ACC in minor salivary gland had the higher propensity for metastasis and poorer prognosis than in major salivary gland [33]. Our data showed that ACC patients with minor salivary gland expressed more WIP1 expression than with major salivary gland. 3) The solid subtype has been shown to have the worst prognosis than the cribriform and tubular pattern [34]. The present study showed that the positive expression of WIP1 in the solid pattern of ACC was much higher than the cribriform and tubular pattern (Table 1). 4) We also showed that the patients with positive WIP1 had a poorer prognosis than with negative. The data are supported by findings of other authors such as Satoh et al [35] reported that PPM1D was a prognostic marker in lung adenocarcinoma patients and Castellino et al [36, 37] showed that high PPM1D expression was correlated with poor prognosis in patients with pancreatic neuroendocrine tumors and medulloblastoma. This indicated that WIP1 expression may associate with the malignant progress of ACC, which validated the data of the experiments *in vitro* that inhibition of WIP1 expression promoted the migration and invasion of ACC cells.

Taken together, in this study we showed that WIP1 functioned as a potential prognosis target in ACC management, particularly cancer metastasis. Much work should be done to look for the precise signal transduction pathways that mediated WIP1 regulation of MMP-9 and VEGF-C in the future. This will help to understand the roles of WIP1 in cancer metastasis and open new doors to treating ACC.

## MATERIALS AND METHODS

### Ethics statement

The study of human specimens was approved by the Institutional Ethics Committee of the West China Medical Center, Sichuan University, China (No.11–032). Every patient signed separate informed consent forms for sampling and molecular analysis.

All animal studies were reviewed and approved by the Animal Care and Use Committee of the West China Medical Center, Sichuan University, China (No.11–343).

### Patients and specimens

Paraffin embedded sections of 121 ACC patients, 20 of pleomorphic adenoma, and 10 of normal salivary gland (from the benign salivary tumor patients) were obtained from the Department of Oral and Maxillofacial Surgery, West China Hospital of Stomatology, Sichuan University between 1996 and 2005 (Patients who had received preoperative chemotherapy, hormone therapy or radiotherapy were excluded). Every patient signed separate informed consent forms for sampling and molecular analysis. This study was approved by the Institutional Ethics Committee of the West China Medical Center, Sichuan University, China. The principal clinical and pathologic characteristics of the patient cohort are summarized in Table 1, followed as previously described [38].

### Immunohistochemistry (IHC)

IHC was performed on 4-mm-cut representative sections by the streptavidin-peroxidase method followed as previously described [39].

### Cell culture

ACC cells lines, ACC-2 and ACC-M, were obtained from the State Key Laboratory of Oral Disease, Sichuan University.

### Cloning, lentivirus preparation, and plasmids

The Lenti-X shRNA expression system (Clontech) was used for the construction of the lentiviral expression construct according to the manufacturer's instructions. Short pairs of sense and antisense DNA oligo encoding a sense-loop-antisense sequence to WIP1 genes were synthesized for the validated corresponding siRNAs, and sequences were as follows: sense strand 5′-CCAAUGAAGAUGAGUUAUAdTdT-3′, antisense strand 3′-dTdTGGUUACUUCUACUCAAUAU-5′, and target sequence CCAATGAAGATGAGTTATA.

The full-length cDNA of WIP1, MMP-9 or VEGF-C was cloned into the pcDNA3 plasmid vector and transfected into cells by LipofectAMINE reagent (Invitrogen) according to the manufacturer's instructions. Stable transfected cells were selected in 400 μg/mL Geneticin and further subcloned.

### Western blot

Thirty-microgram proteins from each sample were separated on 8% SDS-PAGE and transferred electrophoretically to polyvinylidene difluoride membranes (Millipore). Membranes were blocked with 2% bovine serum albumin in TBS containing 0.1% Tween20 (TBST) at 37°C for 2 hours and then incubated for 2 hours respectively with primary antibody. Bands were scanned using a densitometer (GS-700, Bio-Rad Laboratories), and quantification was done using Quantity One 4.4.0 software.

### Quantitative real-time reverse transcriptase-PCR

Total RNA was isolated with TRIzol reagent (Invitrogen) and treated with RNase-free DNase I (Takara) to avoid genomic DNA contamination. PCR amplification of the cDNA template was done using Thunderbird SYBR qPCR mix (TOYOBO) on ABI PRISM 7300 sequence detection system (Applied Biosystems).

### Cell proliferation

The cell proliferation was quantified by the colorimetric MTT assay.

### Wound-healing assay

Cells were plated in 6-well plates at 2.0 × 10^5^ cells/well. When cells reached 80% confluence, the individual wells were wounded by scratching with a pipette tip and incubated with medium containing no FBS to 0, 18 h. Cells were photographed under phase-contrast microscopy (×100) as previously described.

### Transwell invasion assays

*In vitro* cell invasion assays were performed with QCM− 96-well cell invasion assay kit (Chemicon International, Temecula, CA, USA). 5 × 10^4^ cells were seeded into the top chamber coated with Matrigel (BD Biosciences). Complete medium was added to the bottom wells to stimulate invasion. After cells were incubated for 24–48 h, they were stained with 0.1% Crystal Violet. The cells that had invaded through matrigel and reached to the reverse side were counted under a microscope in five pre-determined fields at a magnification of × 400. Each assay was performed in triplicate.

### Xenografts in nude mice

The nude mice (female, 6 weeks of age) were obtained from the Laboratory Animal Center of the West China Medical Center, Sichuan University (Chengdu, Sichuan, China) and kept in a room at a constant temperature (23 ± 2°C) and humidity (50–70%) with a 12-hr light-dark cycle. After 1 week of breeding, fifteen-five mice were randomized and divided into three groups (shRNA-neg, shRNA1 and Control), five mice each. Lentivirus-transfected cells were then injected s.c. (5 × 10^6^ cells/200 μL PBS/mouse) on the back of nude mice. Tumor size was monitored by measuring diameters using vernier caliper weekly, and was calculated as πls^2^/6, where *l* = long side and *s* = short side as described in detail previously [40].

### Statistical analysis

The association between WIP1 expression and clinicopathological variables was compared by the Chi-square test. Overall survival rate was estimated using the Kaplan-Meier method and difference between groups was compared according to the log-rank test. A Cox proportional hazards model was applied to identify prognostic variables that predict overall survival. All statistical analyses were done by the SPSS package (version 13.0).

## SUPPLEMENTARY FIGURES


