# Supplementary material for: WIP1 stimulates migration and invasion of salivary adenoid cystic carcinoma by inducing MMP-9 and VEGF-C
Source: Oncotarget. 2015 Mar 5;6(11):9031–44. doi: 10.18632/oncotarget.3320 (PMC4496200; doi:10.18632/oncotarget.3320)
Supplement: Supplementary file 1 [file oncotarget-06-9031-s001.pdf]

SUPPLEMENTARY FIGURES

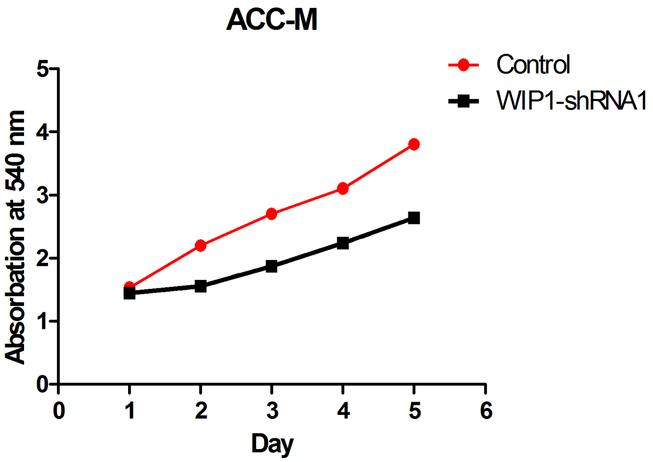

Supplementary Figure S1: Proliferation of ACC-M cells expressing control and WIP1-shRNA1 were measured using MTT assays. The results showed that WIP1 silencing decreased the proliferation rate of ACC-M cells slightly compared with control cells ($p < 0.05$).

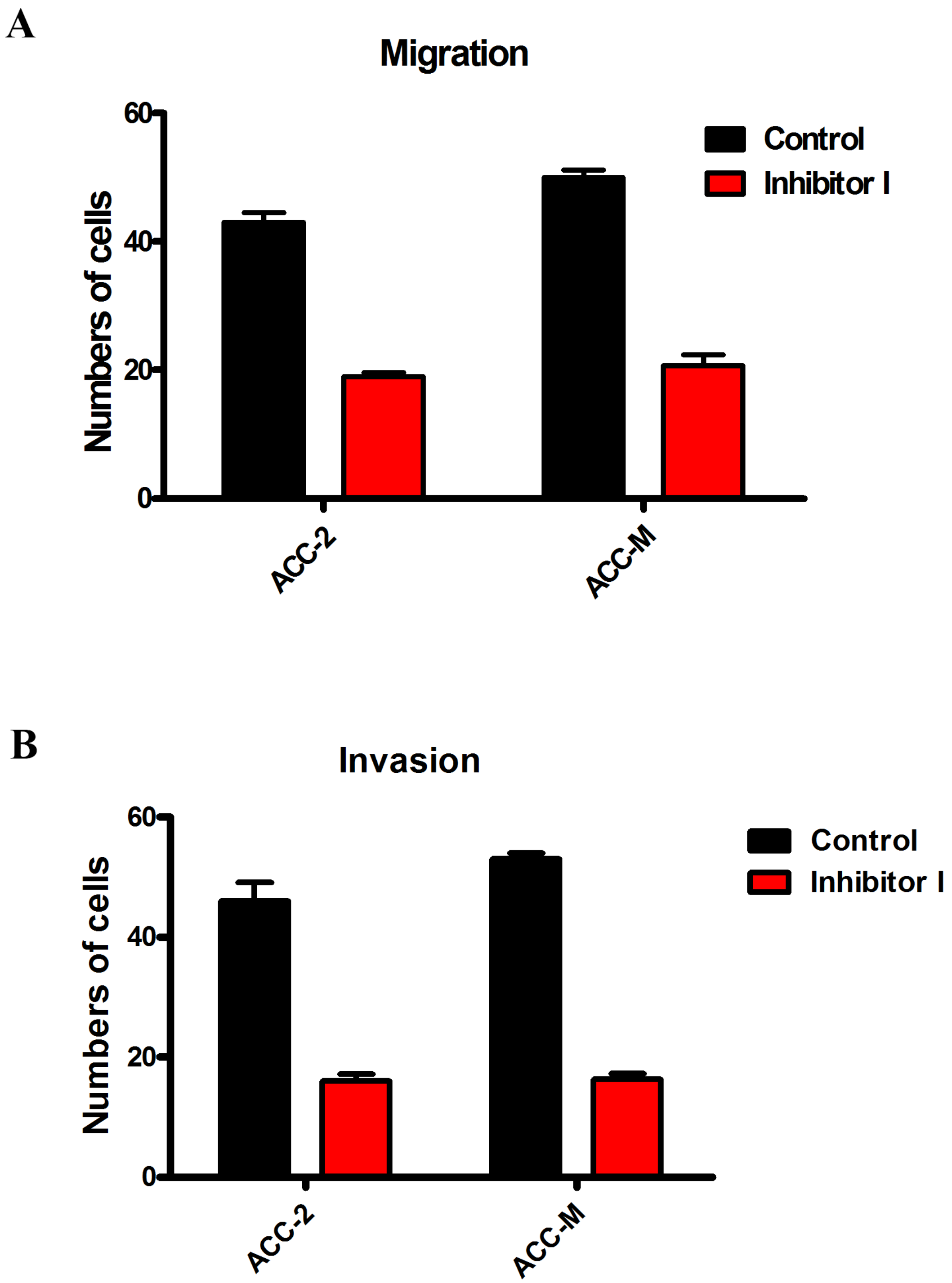

Supplementary Figure S2: The inhibitor I inhibits ACC-M and ACC-2 cells migration and invasion. A and B, Migration **(A)** and invasion **(B)** assays in ACC-M and ACC-2 cells. The mean was derived from cell counts of 5 fields, and each experiment was repeated 3 times.The result showed that the inhibitor I inhibitors significantly blocked ACC-M and ACC-2 cells migration and invasion ($p < 0.05$).
